# Design framework to develop sustainable innovations for addressing One Health challenges

**DOI:** 10.1016/j.onehlt.2025.101031

**Published:** 2025-04-15

**Authors:** Avis Anya Nowbuth, Vikram Singh Parmar

**Affiliations:** a Department of Neuromedicine and Movement Sciences, Norwegian University of Science and Technology, Trondheim, Norway; b Pan-African Organisation for Health Education and Research, MO, USA; c Department of Design, Norwegian University of Science and Technology, Trondheim, Norway

**Keywords:** Framework, Transdisciplinary, One health, Antimicrobial resistance, Design, Intervention development

## Abstract

The complexity of global health challenges requires integrated approaches that crosses traditional boundaries. One Health (OH) offers a holistic approach to address health issues at the crossroads of human, animal, and environmental domains. Antimicrobial Resistance (AMR), a prime example of a cross-sectoral issue and OH challenge, highlights the need for coordinated interventions that consider multiple stakeholders. Current approaches to improve OH challenges and AMR have limited success, often due to a lack of a structured theoretical approach that informs the design and development of solutions for long-term sustainability. Existing frameworks focus primarily on human or veterinary sectors in isolation, leaving a gap in comprehensive, integrated approaches that align with OH principles. The proposed framework addresses this gap by offering a structured approach to both implementing and evaluating interventions that consider all three sectors.

This paper proposes the HEARTf of OHC (*Holistic Engagement and Adaptive Responses Theoretical framework of One Health challenges*), a user-centered design approach aimed at developing sustainable and innovative OHC interventions. The HEARTf of OHC integrates knowledge from social sciences, computer science, industrial design, pedagogy, and health sciences to create context specific solutions that address the specific needs of end-users in the human, animal, and environmental sectors. Additionally, this paper reviews existing frameworks addressing AMR, identifies limitations and outlines the need for a transdisciplinary approach when tackling OHC. By emphasizing the importance of the user-centered design, transdisciplinarity, and continuous evaluation, the HEARTf of OHC aims to bridge the gap between current strategies and improve the development and evaluation of innovative solutions or services. The HEARTf of OHC is a generalizable framework for the design, implementation, and evaluation of OH interventions, where we use AMR as a case study to demonstrate its application.

## Highlights

• The HEARTf of OHC is a generalizable framework for the design, implementation, and evaluation of OH interventions.
• HEARTf of OHC integrates theories from social sciences, computer sciences, industrial design, pedagogy and health sciences.
• HEARTf of OHC uses transdisciplinarity, and includes disciplines that were previously excluded from theory.
• HEARTf of OHC emphasizes the importance of user-centered design, transdiciplinarity and continuous evaluation.
• This framework uses AMR as a case study to demonstrate its application.

## 1 Introduction

The One Health (OH) framework is a collaborative and transdisciplinary approach that recognizes the interconnectedness of people, animals, plants, and the environment to achieve optimal health outcomes [1]. OH has various challenges (OHC) including antimicrobial resistance (AMR), zoonotic diseases, and food safety and security; and existing approaches aim to prevent and control these OHC through collaboration across sectors responsible for human, animal and environmental health [2]. A prominent OHC is AMR, which poses a significant threat to global health [3]. In 2019, it was estimated that 1,27 million deaths were reported as a result of antibacterial resistance (ABR) [4]. It has been predicted that by the year 2050, approximately 39 million people will die as a result to AMR [5]. While AMR occurs within microbes, most drivers of AMR have a human behavioral component [6].

Digital solutions such as educational interventions play a key role in operationalizing the OH approach, specifically for challenges like AMR [[7], [8], [9]]. Despite significant investments and several interventions aimed at combating AMR, this issue remains a serious challenge in global health [7,9]. AMR demands a comprehensive and transdisciplinary approach, incorporating insights from human, animal, and environmental sciences to address it effectively [3]. Understanding the needs and contexts of end-users is essential when developing interventions for OHC, ensuring that these solutions are practical, sustainable, and culturally appropriate. Educational programs that target healthcare providers, veterinarians, and environmental scientists are essential for promoting a shared understanding of OH principles and driving behavioral changes that reduce the spread of AMR [9]. Currently seen within AMR, in the human sector, behaviors such as over- and inappropriate prescribing of antimicrobials, poor infection prevention and control measures in healthcare facilities, and a lack of knowledge about AMR contribute to its spread [10,11]. The use of antimicrobials in animal husbandry, [10,12] and the environmental pollution from inadequate treatment of residential, industrial, clinical and farm waste expands the reach of the resistant bacteria in the environment, contribute to the spread of AMR [10,13]. Addressing AMR calls for a change of behaviors and practices of those who prescribe antimicrobials [6,14]. As a result, there is an increase in research that has begun to explore interventions to reduce AMR from the anthropological and social perspective [6,[15], [16], [17], [18]].

While there is a growing emphasis on OH, current frameworks for intervention, design and evaluation in AMR remain siloed, focusing either on human health or veterinary practices, and oftentimes neglect environmental factors and cross-species transmission [19]. In reviewing frameworks addressing AMR, we found different strategies for intervention, policy development, and evaluation protocols. For example, the University of Leeds established CE4AMR to support projects addressing AMR via community engagement approaches [6]. This framework highlights the importance of delivering behavior-change interventions at the community level while avoiding overwhelming communities. It proposes a 10-step process for developing contextually appropriate and evidence-based content for community engagement, emphasizing the integration of academic evidence and community lived experiences to ensure a comprehensive understanding of the focal problem [14]. Similarly, the framework proposed by Mitchell et al., (2022) emphasizes the need for interdisciplinary and OH collaboration, aligning research activities with global AMR guideline [20]. The framework identifies eight key themes that consolidate priority areas for AMR research. [20] Van Katwyk et al. (2020) proposes a framework for planning, conducting, and disseminating research on AMR policy interventions. [21] Khurana et al., (2023) provides a framework focusing on implementing evidence-based interventions; emphasizing the need for tailored, context specific, cost-effective, and sustainable interventions [22]. The framework highlights the importance of addressing resource constraints, improving coordination mechanisms, and enhancing technical capacity to adapt interventions to local contexts. The AMR-Intervene framework by Léger et al. (2020) describes the design, implementation, assessment of interventions to approach AMR; and the need for coordinated actions across the OH sectors [23]. Despite the growing number of AMR interventions, particularly in the areas of stewardship programs and infection prevention, there is limited evaluation of their effectiveness. While these frameworks offer valuable insights, gaps remain in terms of long-term sustainability and the adoption of these interventions within local contexts. Most of these frameworks, although promising, have been evaluated in one setting, in a controlled setting, or were pilot programs that were not upscaled. Existing frameworks often fail to provide a comprehensive approach that integrates OH considerations or lacks the necessary specificity to guide different types of interventions. Current interventions lack monitoring of their effect on behavior, undermining future prescribers' understanding of social norms and attitudes towards AMR [[23], [24], [25]]. In Nowbuth et al. (2023) systematic review, it was reported that innovative approaches addressing AMR through gamification, as an example, has not been scaled up or designed in a way that allows for them to be accessible across different contexts [24]. Furthermore, without longitudinal studies, it is difficult to understand whether initial successes in changing prescribing behaviors can be maintained over time, leading to the need for addressing the how, why and when interventions are adopted [26]. The failure to include key stakeholders across different contexts in the development and implementation phases reduces the potential for novel innovations to be applicable [27]. Notably, many initiatives do not adequately consider the end-users during the intervention design process [28]. This may result in solutions that are misaligned with users' realities, preferences, and time, leading to a lack of practicality and appeal. Notably, there is limited input on the design aspect of these interventions with little to no data available on the influence of design actors and frameworks [28].

As such, Information and Communication Technology (ICT)-based interventions play a central role in addressing knowledge gaps and monitoring and evaluating the impact, evidenced by literature [29,30]. ICT is a broad term that covers a variety of technologies and tools used for communication and information processing, and provides dynamic interaction, which can be continuously updated and modified to meet evolving needs; they also have a wider reach through smartphones compared to traditional mediums like paper, physical reports, and pamphlets [31,32].

To address these gaps, we propose a novel generalizable framework for the design, implementation, and evaluation of OH interventions. While AMR provides a focused example of how cross-sectoral interventions can be applied within the OH paradigm, the principles outlined in the framework are adaptable to a wide range of OHC, since a transdisciplinary approach is essential for addressing complex OHC. Building on *A Multidisciplinary Approach to ICT Development* [28], we recognize the need for a similar approach in addressing AMR as an OHC. OH inherently spans three domains, and each domain bringing unique insights crucial for developing comprehensive solutions to AMR [10,33]. By applying a user-centered development approach and measuring the adoption of new interventions, we aim to create a structured approach to evaluate and learn from previous projects. Each domain, from healthcare settings to agriculture, has unique characteristics, challenges, and stakeholders [13,34]. Understanding the context also helps in identifying the critical areas for intervention and allows for the evaluation of a framework's effectiveness in a specific context with its potential adaptability to other contexts. Without this understanding, it is challenging to compare different models or scale successful interventions across different settings [27,35]. By combining knowledge from social sciences, design, computer science, and other relevant fields, we can create more effective and sustainable interventions.

We propose the *Holistic Engagement and Adaptive Response Theoretical framework of One Health challenges* (HEARTf of OHC), which is divided into two main components: Component A (focused on the user-centered design, implementation and evaluation phases) and Component B (theoretical foundation for these phases). This framework provides a general approach for OHC interventions, we will use AMR as an illustrative example. The HEARTf of OHC will guide the development of a digital solution to increase the knowledge level of final year medical, veterinary, and agriculture students (also known as future antibiotic prescribers) and thereby control AMR. The proposed framework adequately addresses AMR across the OH sectors and is suitable for incorporation in tertiary education institutions. A key feature of this framework is its focus on identifying the appropriate end users and ensuring their active involvement in the intervention design process. It creates opportunities to merge knowledge from diverse scientific disciplines while simultaneously addressing the needs of the end-users in the context of AMR. Building upon well-known theories on behavior change to gain insights about social norms and beliefs related to inappropriate or over-prescribing behavior of antibiotics among health professionals, persuasive technology to guide creation of content and plan user-interaction for higher engagement with interventions, and diffusion of innovation (DOI) to track and guide the process of adoption for long term sustainability. Although this paper demonstrates the application of the framework using a digital educational tool to combat AMR, the framework is designed to be adaptable to any type of intervention, if the intervention addresses an OHC, the framework provides guidance on how to design, implement, and evaluate it, ensuring comprehensive consideration. In the coming sections, we describe different phases of the proposed HEART of OHC framework, and our plans to test the validity of this framework in the context of AMR. The framework presented is generalizable to OHC, but for the purpose of this paper, we focus on digital educational interventions aimed at combating AMR.

## 2 Description of the HEART of OH framework

**Fig. 1:**
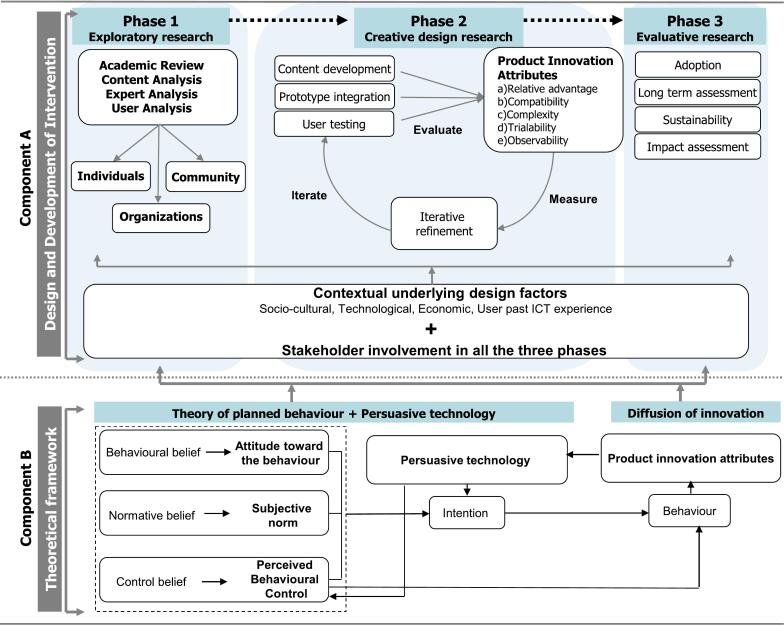
The HEARTf of OHC strategy.

We propose a novel theoretical framework for developing products and service innovations addressing OH challenges such as AMR, food safety, environmental contamination, and climate change. *Holistic Engagement and Adaptive Response Theoretical framework of One Health challenges* (HEARTf of OHC) integrates different methods and relevant scientific disciplines to support the main stages off the development process (Fig. 1). Adapting from *A Multidisciplinary Approach to ICT Development* [28] that has been implemented in the resource constraint environment and have shown effective adoption and mapping of behavior change in the context of public health, [36,37] HEARTf of OHC builds upon this approach to gain insights about OHC and designing sustainable innovative solutions.

The HEARTf of OHC strategy to develop sustainable and innovative solutions involves a deep understanding of end-user needs and current behaviors, derived from field studies with stakeholder (Fig. 1). This approach prioritizes adapting systems to how users can, want, or need to work, rather than forcing users to change their workflows [[38], [39], [40]]. This framework integrates knowledge from multiple scientific disciplines to address the complex challenges of OH. As a pilot, we will be using AMR as a case study. The transdisciplinary approach emphases the necessity of integrating various disciplines which have been identified as critical for addressing OHC [41]. The integrated insights come from the fields of a) social sciences, for understanding societal issues, social norms, and beliefs at community and individual levels to inform the context specific designs of interventions; b) design and computer science, for facilitating the creation of innovative products or services, ensures positive user interaction regardless of users' diverse educational backgrounds; and c) Diffusion of Innovation theory monitors the adoption trends of new innovations among stakeholders to ensure the long-term sustainability of solutions. Studies emphasize the value of a transdisciplinary approach in building a strong workforce capable of tackling complex health challenges effectively, highlighting the importance of communication, teamwork, and interdisciplinary skills [1,33].

HEARTf of OHC is divided into two components: Component A (Design and Development of Intervention) and includes three phases: a) exploratory research, b) creative design research, and c) evaluative research. These stages enable empirical validation of user needs and the development approach. Component B (Behavioral Theories) supports the design framework by applying various theories to guide the development of innovative solutions. Component B explains the selection and application of theories that have been adapted to guide the design and development cycle followed in Component A. Both components are interlinked, with Component B providing the theoretical foundation to the various stages in the user-centered cycle, as defined in Component A. Since both components are interlinked, the theories inform development of innovative solutions and creates opportunities to track reasons for change, impact, and further development. Moreover, the learnings contribute to the existing body of literature from OHC perspective.

### 2.1 Component A: design and development of intervention

(a) Exploratory Research Phase: builds a robust research foundation by understanding target population perceptions, motivations, and barriers related to OHC, in this case AMR. This involves academic review, content analysis, and expert analysis, combined with stakeholder engagement at various levels to gather insights. The exploratory phase identifies target user groups, user characteristics, and social norms. From this phase, the design brief for the intervention, confirming information/ content and user interaction requirements is attained, while relying on the insights and outlines of the stakeholders. Exploratory research involves the following:
  i. Academic Review: Comprehensive review of existing literature on the OHC, in this case: AMR. This helps in understanding the current state of knowledge on a local and global level, identifying gaps and limitations of existing interventions, and highlighting factors that contribute to successful strategies and interventions from other contexts.
  ii. Content Analysis: Critical evaluation and interpretation of existing educational content. For our context, educational curricula that currently addresses AMR within higher education institutions, the strengths, and weaknesses of current materials and how they can be improved or adapted for the target population.
  iii. Expert Analysis: Conducting interviews and consultations with stakeholders and experts. In the case of AMR, this includes healthcare professionals, veterinarians, microbiologists, pharmacists, auxiliary healthcare hospital actors, public health officials, and pedagogical leaders who can provide insights into the specific challenges and needs related to AMR education.
  Overall, this phase involves user observation, focus groups, contextual inquiries, structured interviews, and baseline information surveys (knowledge's, attitudes and perceptions or practices of a user-group). These methods help gather up-to-date and comprehensive data to inform the design process, thereby making the intervention tailored to meet the needs of the end-user and within their context. These insights will be used to create a design brief that confirms the information content and user interaction requirements, which will be used in the (b) creative design research phase.
(b) Creative Design Research Phase: develops context-specific interventions that address OHC by creating, implementing and iterating prototypes based on end-user feedback.
  i. Content Development and Implementation: develop content based on (a) exploratory phase findings, and embed it into prototypes, thereby ensuring coherence and relevance. As part of this phase, there is also a focus on the deployment of the intervention. In the implementation of our case study on AMR education, this includes selecting key themes and topics that address the OHC of AMR [14,21,35]. In the context of addressing AMR education initiatives, this also encompasses integrating the data into established educational frameworks to structure the content. It is also useful to incorporate this data into diverse multimedia elements such as videos, infographics, interactive modules, and quizzes to enhance learning.
  ii. Prototype Development: using the user-centered design principles and behavior theories, a prototype can be developed (Prototype v1). Additionally, the integration of behavioral theories to inform the design of interactive elements that promote behavior change is necessary at this step.
  iii. User Testing: conducting usability testing and heuristic evaluations to gather feedback and refine the prototype/ to further develop it into v2 (Prototype v2). Under this phase, evaluating the effectiveness of the content through pre- and post-tests, surveys, and observational studies to iteratively refine the content will be useful.
  iv. Iterative Refinement: Based on the user-testing results, we can continuously improve prototypes thereby improving functionality and user experience and tailoring it to the target population and context.
  This phase involves designing the intuitive and user-friendly interfaces, implementation of the intervention within the field so that the intervention can be adapted to local contexts and operationalized according to eh pre-established design, further development the intervention by including quizzes, simulations, and feedback mechanisms to gather information in the early stages of the prototype/ v1. Lastly, this phase embeds the educational content into the prototype for seamless integration. Continuous monitoring during this phase allows for real-time adjustments, ensuring fidelity to the intervention plan before moving to the evaluative phase. By focusing on comprehensive content development, and iterative refinements of prototypes, this stage ensures that the intervention is grounded in theory, but can be designed to be also practical, engaging, and effective in the real-world setting. This approach improves the chances of sustainable adoption and meaningful impact in addressing an OHC.
(c) Evaluative Research Phase: assesses the effectiveness and adaptability of interventions, ensuring readiness for real-world application.
  i. User Adoption: by conducting longitudinal studies to measure user adoption and compare baseline data with the intervention data.
  ii. Innovation Attributes: additionally, evaluating the intervention based on product innovation attributes from diffusion theory (a) relative advantage, (b) compatibility, (c) complexity, (d) trialability and (e) observability [42,43]. In the context of AMR education, these innovation attributes help in tailoring the content and delivery methods to resonate with the target audience [42,43]. In the context of AMR education, (a) relative advantage refers to the perceived benefits- of the educational intervention over existing methods. The advantages of the new intervention will be compared against existing interventions, for example, if the new intervention (gamification) uses interactive media that makes complex information easily understood then it is considered to have a significant advantage. The concept of (b) compatibility measures how well an intervention fits with existing values, experiences, and needs of the target audience. For AMR education, this could mean aligning the content with the local beliefs and practices around prescribing antimicrobials. When designing an intervention, (c) complexity plays a role in how the intervention is adopted. For AMR education interventions, the intervention should be straightforward, avoiding medical jargon, and presented in a local language. Allowing users to experiment with the intervention addresses the (d) trialability, where being able to trial the intervention before fully committing to the new intervention can increase adoption. For AMR education, this involves piloting the program in selected pilot sites before a wider rollout. Lastly, in addressing (e) observability, the benefits of the intervention should be visible to potential adopters, i.e. the extent to which the innovation provides tangible results. In addressing AMR education, this might be achieved through visible improvements in the community health practices or testimonials from the early adopters. Additionally, observability allows for organizations and policy makers to assess the tangible benefits and impact of the innovation, thereby facilitating informed decision-making [44]. Projects that utilize this framework can benefit from a more comprehensive understanding of end-user needs and contextual factors since it incorporates end-user involvement, making these projects better align with real world applications.
  Overall, this phase uses thematic and qualitative studies, heuristic testing, and implementation research frameworks to gather and analyze user feedback. Iterative improvements based on feedback to optimize interventions for real-world contexts, enhancing sustainability and effectiveness. From here, we suggest a continuous gathering of user feedback and data to inform ongoing improvements and adaptations of the prototype.

### 2.2 Component B: theoretical framework

We are proposing the combination pre-existing, validated theoretical frameworks to approach the development of an intervention primarily due to the multidisciplinary aspect of OHC. We use the Theory of Planned Behavior (TPB), Persuasive technology (PT) and Diffusion of Innovation (DOI).

(a) The use of theories is integral to inform and test interventions for expanding basic science and for the development of interventions that have real-work practical utility [45]. Understanding societal dynamics using the Theory of Planned Behavior (TPB) is central in understanding societal issues, norms, and beliefs at community and individual levels to inform intervention design [46,47]. The TPB was chosen as a guiding behavioral theory for this framework due to its robust capacity to predict intentional, health-related behaviors (for example, in the context of AMR, the compliance with stewardship practices). Unlike the self-determination theory (SDT), which emphasizes internal motivation, TPB offers a more practical approach to understanding how attitudes, subjective norms, and perceived behavioral control directly influence specific behavioral outcomes in OH interventions [[46], [47], [48]]. Additionally, we found that oftentimes in studies, there is an integrated model of which SDT feeds into TPB, or the need for a deeper analysis on how SDT variables interact with or help implicit influences and structural barriers [[49], [50], [51]].
  The TPB explains the intentions when engaging in a particular intervention are influenced by three key factors: *(a.i) attitudes towards their behavior*, *(a.ii) subjective norms,* and *(a.iii) perceived behavioral control* [46,47]*.* These three factors together shape an individual's behavioral attention [46,47]. In the context of AMR education, educational interventions can be designed to change prescribers' behaviors regarding prescription practices while they are students.
  When considering the *(a.i) attitudes towards their behavior*, we consider the beliefs about their prescribing behaviors and the related outcomes. A positive attitude is the prescription of antimicrobials only when indicated will effectively combat AMR and improve patient outcomes. A negative attitude is when the prescribers believe that not prescribing antibiotics might upset the patients, lead to patient dissatisfaction, or lose patients to other medical doctors or veterinarians who will prescribe what the patients want. Using this in developing interventions for AMR helps promote positive attitudes towards responsible prescribing, which encourages prescribers to follow best practices and use antibiotics more wisely. This could be by presenting case studies, and patient stories to create an emotional connection, highlighting successful outcomes of responsible prescribing through data and real-life examples, and the use of interactive and engaging educational formats to reinforce the positive impact of responsible antimicrobial use.
  When considering the *(a.ii) subjective norms* factor*,* we consider the perceived social pressure to perform or not perform a specific behavior [47]. This is influenced by colleagues, patients, guidelines, and intuitional and governmental policies. A positive norm, for example, is colleagues and healthcare institutions encouraging and support responsible prescribing; while negative norms is the cultural of over-prescribing due to patient demands or peer pressure. Applying this concept to AMR education involves promoting healthcare professionals as champions of responsible prescribing, making patient education materials available in clinics, and using posters or reminders to support hospital guidelines.
  When considering the *(a.iii) perceived behavioral control*, relates to the prescribers' perception of their ability to perform a behavior, influenced by knowledge, resources and time [47]. High control is considered when prescribers have better confidence in diagnosing infections accurately, thereby making appropriate prescribing decisions; while low control results when the prescriber feels pressured by time constraints, lack of access to diagnostic tools or faces pressure from patients to prescribe. A higher perceived control increases the likelihood that prescribers will engage in responsible use of antimicrobials because doctors, dentists, and veterinarians feel more capable and supported in their decision-making. To improve adoption using the perceived control, there is a need to develop and distribute user-friendly decision support tools that integrate with existing clinical workflows, the organisation of regular training sessions and provide easy access to updated clinical guidelines, and the use of data-driven feedback to highlight areas for improvement and celebrate successes in responsible prescribing practices.
  Using the TPB to address AMR education involves understanding *the attitudes, subjective norms, and perceived behavioral controls* in the use of antimicrobials. Overall, when addressing AMR education, developing educational materials that highlights the consequences of inappropriate antimicrobial use by using real-life stories and statistics about the dangers of AMR that creates an emotive response that will encourage a positive attitude towards responsible prescribing [52]. Additionally, addressing the subjective norms by engaging in community leaders, leaders in pedagogy and influencers in the academic and various sectors to be involved in the educational campaigns. This could shift social norms, making responsible prescribing of antimicrobials more understood by the community and prescribers [53].
(b) Persuasive Technology (PT): aims to change attitudes or behaviors through persuasion and social influence rather than coercion or deception [54]. Social cues refers to elements that mimic human social interactions and are integral to this process of persuasion, since they enhance the perceived credibility and attractiveness of technology, thus increasing its persuasive power [54]. Fogg identifies several types of social cues that can be included in the development of an intervention to make it more persuasive [54]. HEARTf further contributes to progressing PT since it builds on already well-established theory by ensuring that the intervention design aligns with user needs and preferences, we can facilitate the development of user-friendly, innovative products or services by incorporating the feedback from the target audience throughout the development process. When addressing AMR education, this can be done by identifying user needs through conducting surveys and focus groups with the various students and educators to understand their specific challenges and needs regarding AMR education. By conducing these user-centered surveys, this could reveal gaps in current strategies used to educate students. Through the development of interactive modules that provide AMR education, we can use iterative testing with end-users to refine these tools. Additionally, adapting educational content to reflect local languages, cultural practices and health literacy levels ensures that the materials are relatable and easily understood by the target audience.
(c) By using the DOI theory to track and improve the adoption of interventions over time, we can monitor the trends among stakeholders to ensure long-term sustainability [42,43]. This can be accomplished in a few ways, namely through the identification and collaboration with early adopters to pilot the AMR educational intervention. Their successful experiences, and testimonials, can serve as case studies to encourage the broader adoption. Additionally, by making the benefits of the intervention visible (showing better knowledge scores for students who have piloted the intervention) will help in the adoption of the intervention. Through the DOI, ensuring that the intervention fits well with existing healthcare practices and sectoral contexts, making it a seamless part of existing structures.

By integrating TPB, PT, and DOI theory, this framework ensures that AMR interventions, and general OHC interventions, are scientifically grounded, user-friendly, and sustainable. This also facilitates the development of practical, engaging, and effective interventions that can significantly contribute to addressing OHC. The proposed HEARTf of OHC emphasizes a transdisciplinary approach, integrating insights from social sciences, design, computer science, and innovation diffusion to develop effective interventions to combat OHC. By focusing on comprehensive content development and iterative prototype integration, HEARTf ensures interventions are made to be practical, engaging, sustainable and suitable for the real-world setting.

## 3 Conclusion

While demonstrated through an educational intervention example, is applicable to interventions across all areas addressing OHC. We presented a review on existing frameworks that address pedagogy, technological design and behavior change models. The user-centered phases - exploratory research, creative design research, and evaluative research - ensure that interventions are tailored to user needs, culturally relevant, and adaptable to real-world settings. By emphasizing the importance of identifying the appropriate end-users and facilitating collaboration across disciplines, the framework ensures that any OH intervention, whether it focuses on education, clinical practice, policy, or environmental management, can be effectively developed and implemented. The theoretical framework underpins these phases with theories from behavioral science and innovation, guiding the development process and ensuring empirical validation. The HEARTf of OHC is currently being used in the development of an intervention addressing AMR. The anticipated impact includes improved user engagement, enhanced effectiveness of intervention and sustainable adoption of AMR solutions.

## CRediT authorship contribution statement

**Avis Anya Nowbuth:** Writing – review & editing, Writing – original draft, Visualization, Validation, Methodology, Investigation, Formal analysis, Data curation, Conceptualization. **Vikram Singh Parmar:** Writing – review & editing, Validation, Supervision, Resources, Project administration, Methodology, Formal analysis, Conceptualization.

## Funding

This research did not receive any specific grant from funding agencies in the public, commercial, or not-for-profit sectors.

## Declaration of competing interest

The authors declare that they have no known competing financial interests or personal relationships that could have appeared to influence the work reported in this paper.

## Data Availability

Data will be made available on request.
